# Testing the thermal properties of modern ventilated facade fastening systems

**DOI:** 10.1038/s41598-023-27748-4

**Published:** 2023-01-18

**Authors:** Mirosław Grabowski, Mieczysław E. Poniewski, Jacek Wernik

**Affiliations:** Faculty of Civil Engineering, Mechanics and Petrochemistry, Warsaw University of Technology, 09-400 Płock, Poland

**Keywords:** Engineering, Mechanical engineering

## Abstract

The study reported in this paper investigated a set of building fasteners used in ventilated facades. For the building fasteners actually present in the industrial market the values of the effective thermal conductivity were measured experimentally. These values were used next in numerical simulations run with COMSOL Multiphysics software application. The validation of the simulation model was done in specific additional experimental test. The paper presents a method of determining the effective thermal conductivity coefficient for fasteners with a novel design. Temperature distributions and heat fluxes were determined for different variants of multilayer walls with the fasteners. The calculation of the effective thermal conductivity coefficient for a structural profile is based on the heat balance of the measuring stand. The performed tests show not only an expected reduction in the coefficient value for structures in which stainless steel is used. The results also demonstrate that the fasteners with holes cut out in their structures have significantly lower effective thermal conductivity coefficients than those with solid walls. This effect can be justified by the formation of labyrinth-like narrowings extending the conductive heat flow path in the fastener. As a final result of the experimental tests and the COMSOL simulations the application of the effective thermal conductivity as the new indicator of a thermal effectiveness of building fasteners is proposed in industrial practice. Consequently the design of the building fasteners with various shapes of holes is recommended for improving their insulation features.

## Introduction

In the past, the criteria for selecting construction fasteners for the construction of a ventilated wall were mainly strength and mechanical properties. After tightening the regulations on the insulation conditions that must be met by the walls of buildings and the high efficiency of materials insulating the surface of the walls, it turned out that the losses for which the heat flux transmitted by the supporting structures, including building fasteners is responsible, is an increasing share in entire heat losses. This situation forces designers to use construction fasteners with better and better insulating parameters. A new solution, not yet widely used, but as this research has shown—the use of extending the heat flow path holes in fasteners is effective. The authors of the article used standard methods to assess the thermal properties of the tested fasteners: experimental tests and numerical simulations.

Numerical simulations and experimental tests are widely used to study the properties of various building fasteners^1,2^ in terms of strength^3,4^ and materials used. Thermal imaging allows the identification of fastener thermal properties^5,6^ and their impact on wall systems with multiple layers. Ventilated facade systems, a desirable solution due to the energy savings they provide^7,8^, are studied using numerical simulations^9^. Studies focus primarily on the effects of seasonal weather on the performance characteristics of a facade^10^ or the influence of ventilated facades on the energy demand of buildings^11^. In^12^, the authors investigated heat transfer coefficients of walls with different quantities of fasteners used to mount the panel on the façade. They found a significant increase in coefficient values of ventilated panels compared to traditional ventilated facade systems. The present article shows that also individual elements of ventilated facades have an impact on heat conduction.

Ventilated facades consist of an interior layer, insulation, ventilation chamber, and exterior finish (outer skin panels). The system reduces the thermal loads due to solar radiation and protects against weather conditions^13^. Today, there is much research on facades being an indispensable element of the high-rise architecture of modern cities^14^. The use of ventilated facades has been analyzed in detail in^15^.

Building fasteners are important elements of ventilated facades and are subject to applicable European^16^ and Polish^17^ regulations. As these regulations provide for a gradual reduction of heat transfer coefficients for building partitions, there is a need for a fastener design that will aid in minimizing the coefficients to the required levels. The requirements relating to thermal insulation, strength, and fire safety are currently satisfied by the following options:

1. Designing fasteners made of materials with a lower thermal conductivity (e.g. stainless steel instead of aluminum alloys);
2. Increasing fastener design complexity (e.g. use of holes extending the heat flow path);
3. Applying pads made of insulating materials.

The third option is difficult to implement if the strength and fire safety requirements are to be satisfied at the same time. The first option yields good results but has severe limitations. A material that will meet future requirements for conductivity coefficient while at an acceptable price is hard to find. The second option is up-and-coming, especially in combination with the first option.

This paper presents the results of experimental and simulation studies of the thermal properties of ventilated façade fastening systems in which the first two options were applied. The parameter characterizing these properties is effective thermal conductivity, defined further in the article. The effective thermal conductivity is mainly influenced by the thermal conductivity of the material from which the fastener is made, its design (e.g. the use of openings impeding the heat flow) and thermal resistance at the contact points of heat transfer surfaces. It should be added that the measurement method applied includes the effects of all the factors listed above. Therefore, the effective thermal conductivity so determined characterizes the actual thermal properties of the fastener.

**Figure 1:**
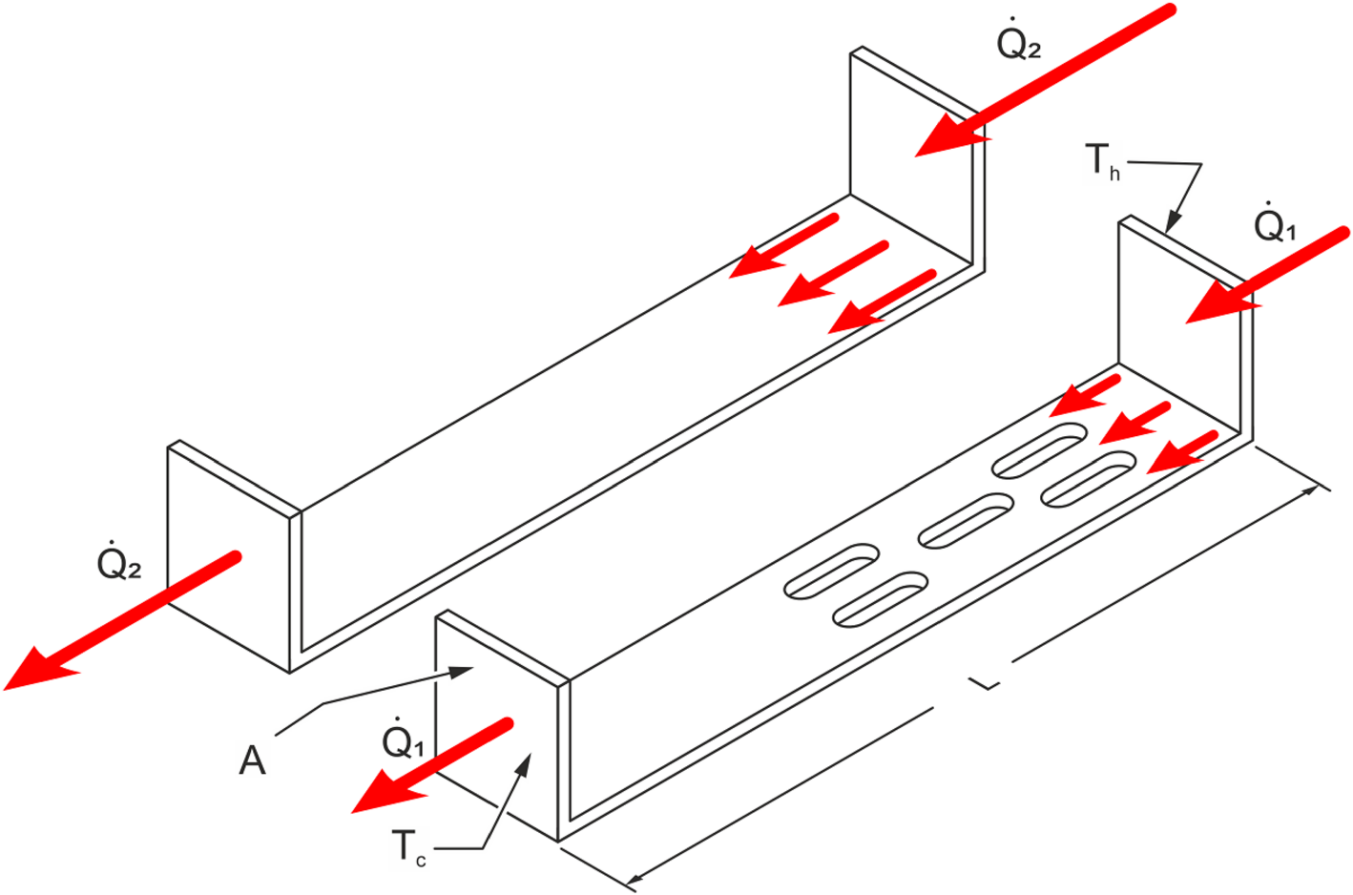
Illustration of the effect of the thermal conductivity of the fastener material and the use of heat flow-impeding openings on the effective conductivity of building fasteners and heat flux transferred. Description: A—heat transfer surface (interface between the fastener and the cooler surface) (m^2^), L—fastener length in the heat flux direction (m), T_h_—average temperature of the heater surface (K), T_c_—average temperature of the cooler surface (K).

Figure 1 illustrates concept of heat transfer coefficient measurement and the effect of individual input factors, such as the use of openings.

## System introduction

**Figure 2:**
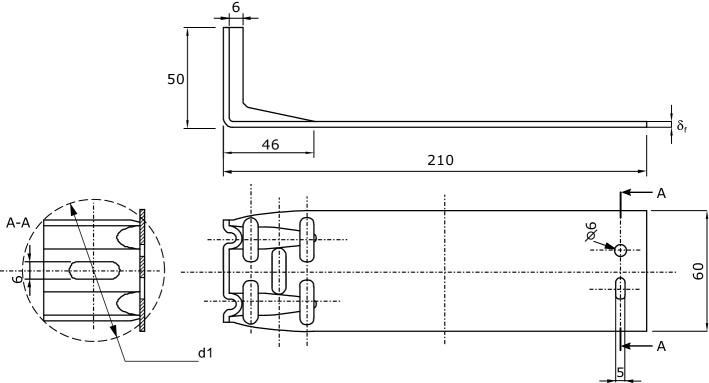
Dimensions of a building fastener for attaching ventilated facades.

This paper reports the study results for currently available fastening systems used to attach ventilated facades. The design of the fasteners combines options 1 and 2 described above. Figure 2 shows the dimensions of the fastener.

**Figure 3:**
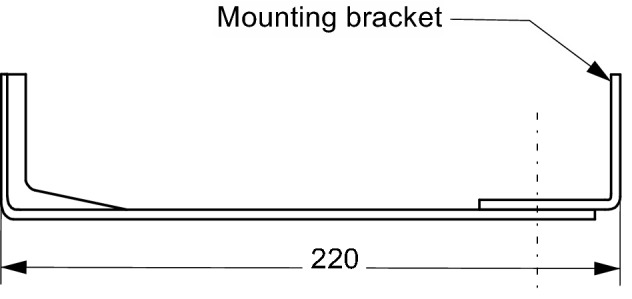
Building fastener with mounting bracket.

Figure 3 presents complete building fastener unit, supplemented with mounting bracket. In the form shown in Fig. 3, the fastener assembly is used to fix the claddings and has been subjected to experimental tests in this form.

## Measurement method

Thermal balance is the primary method to analyze many practical problems of solid body thermomechanics and building physics.

The measurement of the effective thermal conductivity coefficient λ of a building fastener is based on the heat balance of the measuring stand. By neglecting heat losses to the environment, the numerical value of the heat flux supplied by the electric current flowing through the heater equals the numerical value of the heat flux conducted through the surfaces of the fastener. It can be expressed by Eq. (1):

1 $$ \dot{Q}_{el} = \dot{Q}_{cond} $$

where $\dot{Q}_{el}$—electric power supplied by the heater (W), $\dot{Q}_{cond}$—heat flux transferred through the fastener (W).

The stand should ensure the lowest possible heat losses tenvironment to meet the conditions of Fourier’s law of one-dimensional heat conduction. The use of direct current enables a simple and accurate determination of the thermal power supplied by the heater:

2 $$ \dot{Q}_{el} = I \cdot U $$

The heat flux transferred between two flat surfaces and for the relationships given in Fig. 1, can be written in the way allowing determination of the effective thermal conductivity:

3 $$ \lambda^{\prime } = \frac{U \cdot I \cdot L}{{A \cdot \left( {T_{h} - T_{c} } \right)}} $$

where *A*—heat transfer surface (interface between the fastener and the cooler surface) (m^2^), *L*—fastener length in the heat flux direction (m), *T*_*h*_—average temperature of the heater surface (K), *T*_*c*_—average temperature of the cooler surface (K), *λ*—thermal conductivity coefficient for a homogeneous material (W m^−1^ K^−1^) or, as used further in this article: *λ′*—effective thermal conductivity for a non-homogeneous material.

The proposed measurement method determines the actual effective thermal conductivity coefficient that includes the impact of the fastener interior structure (holes extending the heat flow in the heat exchange surface, extrusion) and the influence of thermal contact resistance on its heat conduction capacity.

## Measurement stand

**Figure 4:**
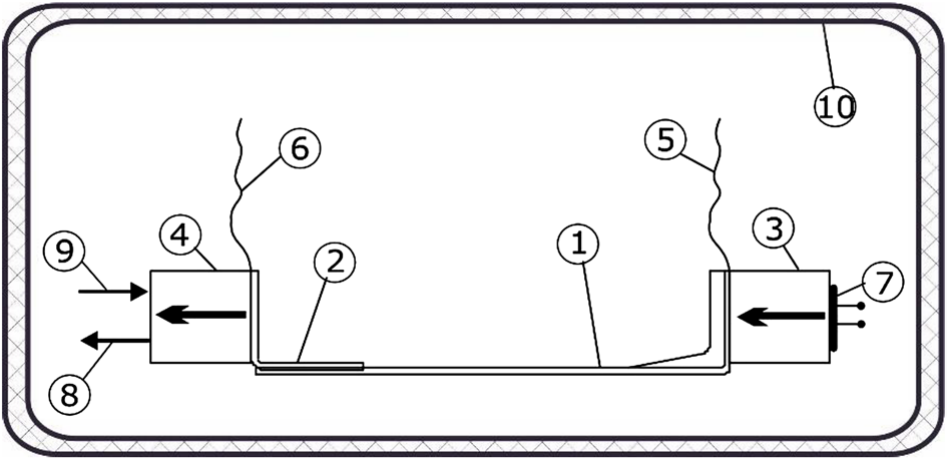
Schematic diagram of the measuring stand. Designations: 1—fastener, 2—mounting element, 3—heater, 4—cooler, 5, 6—K-type thermocouples (0.5 mm in diameter), 7—heating resistor, 8—coolant inlet 9—coolant outlet, 10—insulated casing.

**Figure 5:**
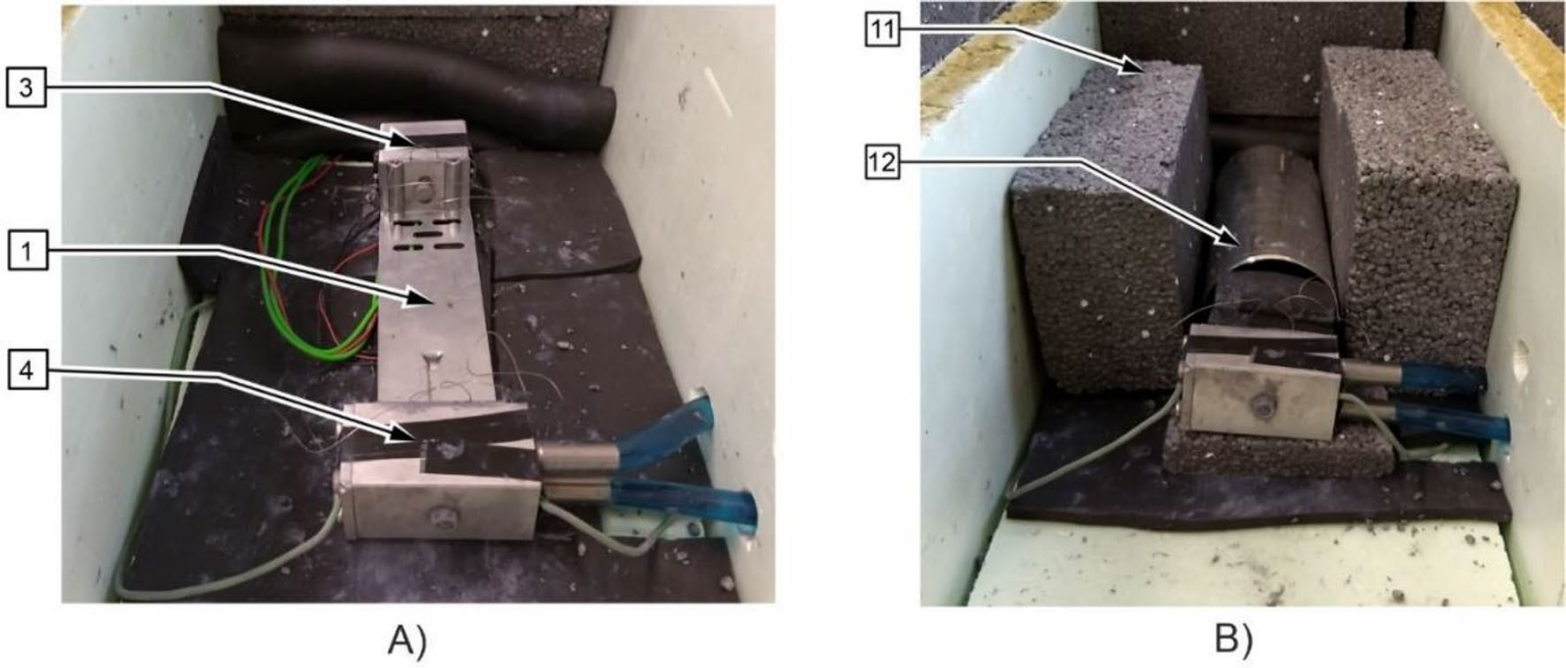
(**A**) View of the experimental section before being insulated and placed in the Dewar flask. Designations as in Fig. 4, (**B**) View of the experimental section after being placed in the Dewar flask and during the insulation process. Designations: 11—Styrofoam insulation, 12—Dewar flask.

The tests included measurements of the effective thermal conductivity coefficient of the building fastener presented in Fig. 2. During the measurements under conditions of actual use, the fastener is supplemented with a mounting bracket (Fig. 3) to which the cladding (actual conditions) or the cooler (measurement conditions) is attached. The surface of the component is the heat-receiving surface. The measuring stand was built to the measurement concept presented in “Introduction” section. The schematic diagram of the stand is shown in Fig. 4, and a view of the measuring section is in Fig. 5. The main part is the assembly connecting the tested fastener with the heater and the cooler. Two miniature thermocouples are placed between the fastener wall and the heater. The cooler is attached to the fastener's opposite end, and two miniature thermocouples are placed between the fastener and the cooler. The heater, fastener, thermocouples, and cooler were coated in silicone to reduce the thermal contact resistance at the interface between two heat-conducting surfaces.

**Figure 6:**
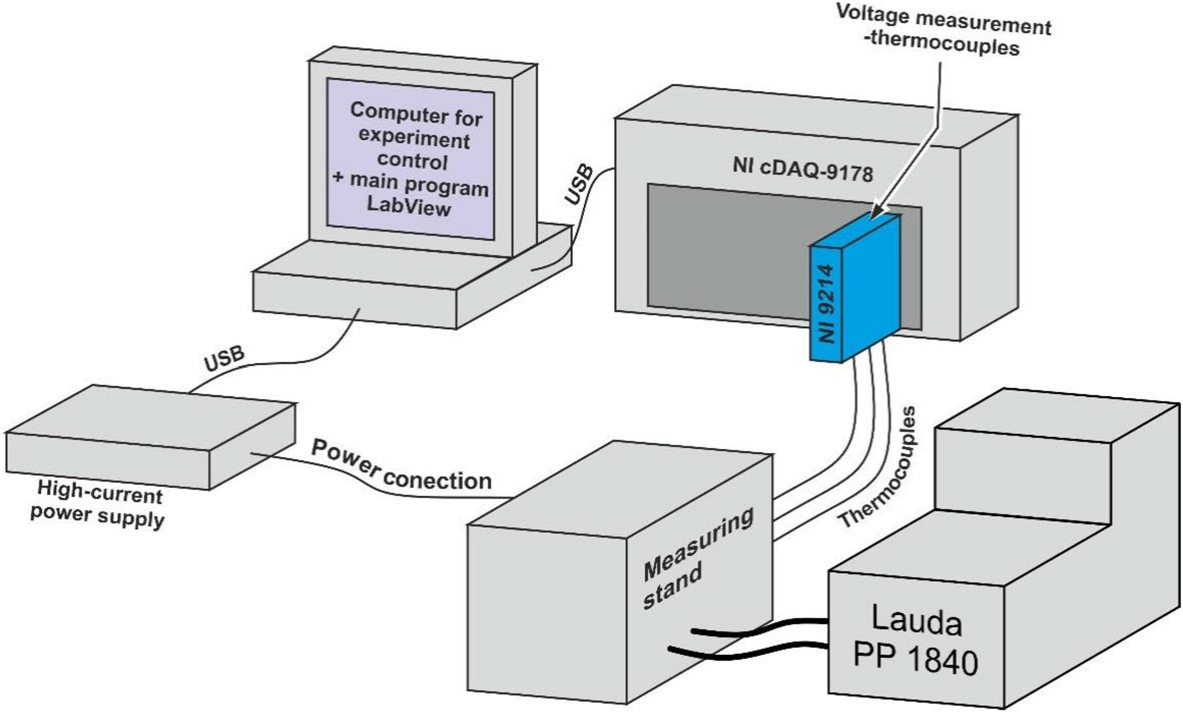
Scheme of the measuring system.

The temperature measuring system consisted of three K-type thermocouples connected to the NI 9211 measuring module and then to the cDAQ-9171 module connected to a PC control (Fig. 6). The script controlling the experiment and data flow was written in LabView. Two thermocouples were placed on the cooling surface and one on the heating surface. The use of only one thermocouple on the heating surface was due to the irregular surface of the fastener. As seen in Fig. 4, the heater consisted of heating resistor 7 and aluminum block 3 for uniform heat distribution. Thermocouples 5 and 6 were placed between the aluminum block and the fastener. The entire temperature measurement path was calibrated using a Testo 735-2 thermometer with a Pt100 Testo 0614 0235 measuring sensor. The measurement was performed under steady-state conditions. As mentioned in the new text, the sample was insulated with a thick layer of Styrofoam and a Dewar flask. The measurement was preceded by a 60 min warm-up period to allow the setup to attain steady state conditions. Then, the actual measurement session comprised 20–30 partial measurements taken every second. The steady state and good thermal insulation of the setup allowed a considerable number of cumulative partial measurements.

**Figure 7:**
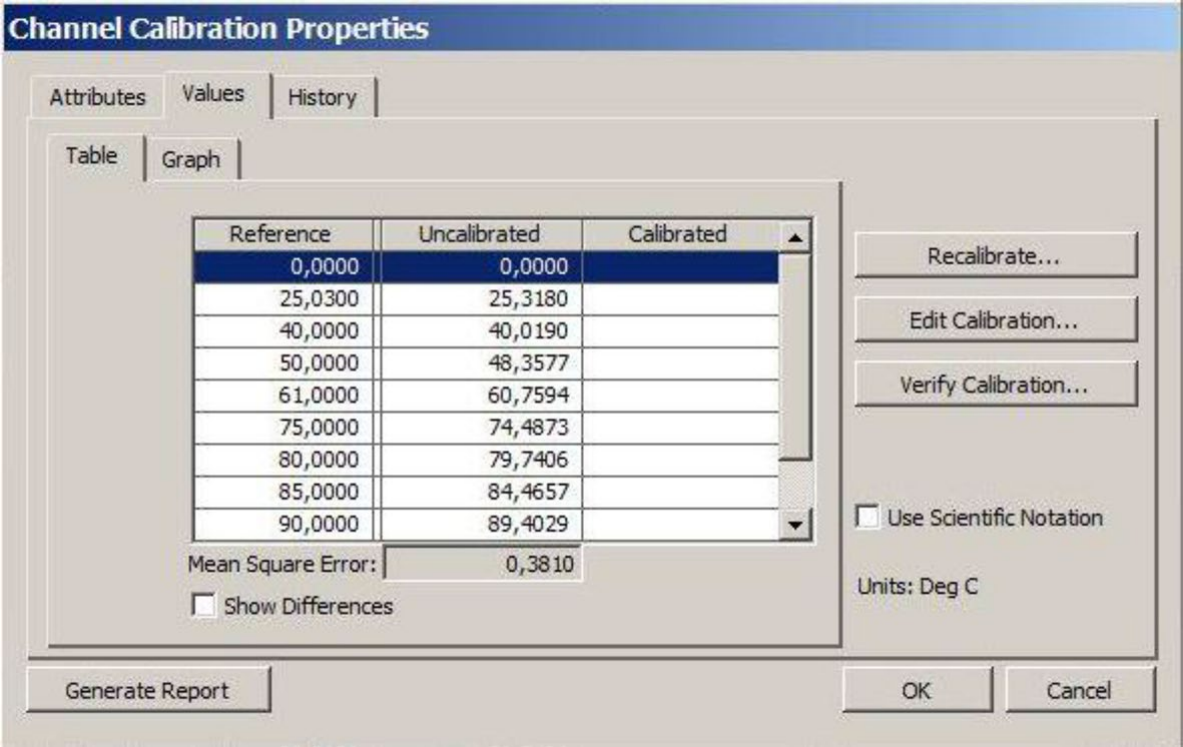
Data for plotting the calibration curve being entered in LabView.

**Figure 8:**
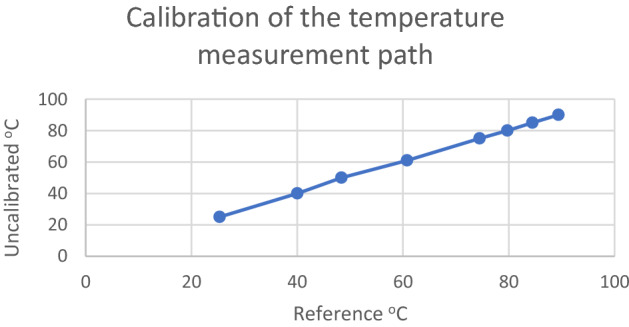
Calibration curve for one temperature measurement path.

In order to obtain a low value of effective thermal conductivity coefficient uncertainty, the measurement system was provided with high accuracy measurement sensors coupled with a computer system for reading and recording data. The LabView environment allowed convenient calibration of the temperature measurement paths, Figs. 7, 8 and Table 1 show an example of a calibration curve for one of the thermocouples in the experimental stand.

**Table 1:** Data from the calibration of one measurement path.

Reference	Uncalibrated	Reference—calibrated
25.030	25.318	− 0.288
40.000	40.019	− 0.019
50.000	48.358	1.642
61.000	60.759	0.241
75.000	74.487	0.513
80.000	79.741	0.259
85.000	84.466	0.534
90.000	89.403	0.597

Calibration of the measurement path consisting of measurement modules by National Instruments and thermocouples K-type by Czaki resulted in the maximum permissible measurement uncertainty of 0.5 K. The calibration covered the range from 20 to 90 °C within which all measurements in the series were made.

## Experimental results

The actual calculations determined the effective thermal conductivity coefficient λ′ according to formula (4). During the measurements, the voltage supplying the heater was approx. 10 V and the current flowing through the heater was approximately 0.6 A. Both parameters were constant throughout the experiment. For experiments and calculations, variants of stainless steel and aluminum alloy fasteners were selected and made according to the solutions of various manufacturers. The outer dimensions of the steel fasteners were 50 × 60 × 210 mm, and those of the aluminum alloy fasteners were 40 × 60 × 210 mm. The steel fasteners had strengthening fins on the side mounted to the wall for added rigidity. In one fastener type, holes were cut out (Fig. 1) to reduce the effective thermal conductivity coefficient. This effect was confirmed in this study. The following fastener variants were investigated:

1. Stainless steel, δ_f_ = 2 mm,
2. Stainless steel, δ_f_ = 3 mm,
3. Stainless steel, 5 holes, δ_f_ = 3 mm,
4. Aluminum alloy, δ_f_ = 2.75 mm,
5. Aluminum alloy, δ_f_ = 4 mm.

The properties of the materials for fastener fabrication are compiled in Table 2.

**Table 2:** Material properties.

Material	Thermal conductivity (W m^−1^ K^−1^)	Density (kg m^−3^)	Heat capacity (J kg^−1^ K^−1^)
Stainless steel 1.4301	15	7900	500
Aluminum alloy AW-2017A	134	2800	873

The calculation results of the fastener average effective thermal conductivity coefficient are presented in Table 3.

**Table 3:** Average effective thermal conductivity coefficient of the building fasteners.

Fastener no	Description	Effective thermal conductivity coefficient The average of all values in measurement series (W m^−1^ K^−1^)	Absolute standard uncertainty (W m^−1^ K^−1^)	Relative combined standard uncertainty (%)
1	Stainless steel fastener, δ_f_ = 2 mm	3.97	± 0.05	1.13
2	Stainless steel fastener, δ_f_ = 3 mm	4.85	± 0.08	1.57
3	Stainless steel fastener, δ_f_ = 3 mm with 5 holes	4.26	± 0.06	1.39
4	Aluminum alloy fastener, δ_f_ = 2.75 mm	15.68	± 0.46	2.91
5	Aluminum alloy fastener, δ_f_ = 4 mm	21.04	± 0.68	3.23

Fasteners no. 2 and 3 have similar mechanical strength and dimensions to fastener no. 5 (Table 3) and can be used interchangeably. It thus seems advisable to identify a fastener with the best thermal parameters and determine its potential for reducing heat losses. Fasteners 2 and 3 are directly comparable due to the identical dimensions, type, and thickness. The Table 3 shows that the holes obstructing the heat transfer reduce the effective thermal conductivity coefficient.

It should be noted that the effective thermal conductivity coefficient is based on Eq. (3) and is related to the contact area of the fastener with the surfaces of the heater and cooler.

Aluminum alloy fasteners have much higher thermal conductivity coefficients and their design differ from those made of steel. The diagram allows another inference about the effect of the fastener thickness, i.e., reduced thickness leads to the reduced effective thermal conductivity coefficient of the fastener unit.

The correctness of the method was verified using the measurement of the thermal conductivity of a homogeneous material—a cuboid 50 × 50 × 100 mm made of AW-2017A (AlCu4MgSiA) aluminum alloy. The obtained result for the chosen alloy was 143.5 W m^−1^ K^−1^, close to the value specified in EN 573-1 for AW-2017A.

## Numerical simulations

The experimental studies were supplemented with numerical simulations aimed at determining the heat transfer coefficient and heat loss from a unit surface (1 × 1 m) of the ventilated wall.

**Figure 9:**
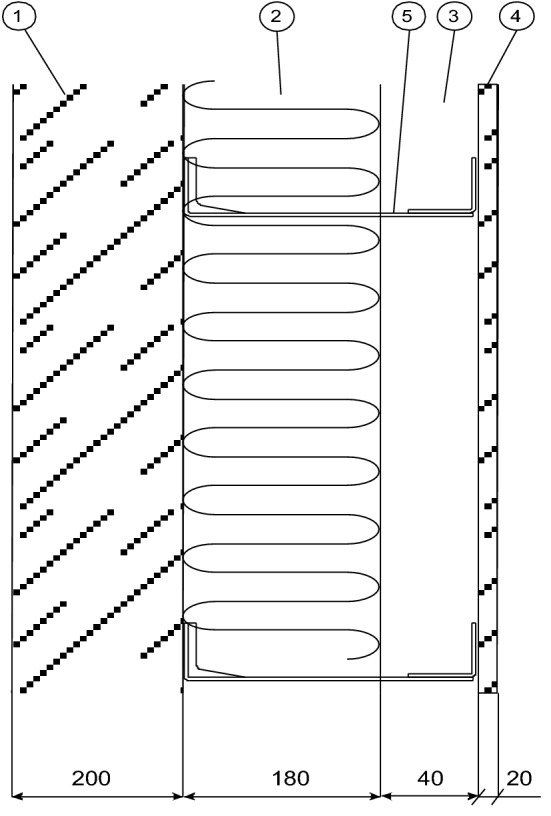
Cross-section of a multilayer wall analyzed via numerical simulation. Denotations: 1—wall (reinforced concrete), 2—insulation (mineral wool), 3—air gap, 4—two variants of external cladding: fiber-reinforced concrete, δ_p_ = 20 mm, or aluminum alloy panels, δ_p_ = 3 mm, 5—fastener.

The simulations were carried out using the commercial COMSOL Multiphysics package^18^. A digital model of the multilayer wall section with the fastener was based on the assumptions in Fig. 9. The simulations were carried out for variants of 1 × 1 m multilayer wall section with the outer cladding made of (a) fiber-reinforced concrete (λ = 1.5 W m^−1^ K^−1^) and (b) aluminum alloy panels (λ = 167 W m^−1^ K^−1^). The following two variants were considered:

- Stainless steel fastener with heat flow extending holes, δ_f_ = 3 mm, λ = 4.26 W m^−1^ K^−1^.
- Aluminum alloy fastener, δ_f_ = 4 mm, λ = 21.04 W m^−1^ K^−1^.

Table 4 compiles the data of the materials used in the multi-layer wall elements included in the numerical simulations.

**Table 4:** Materials data for the multilayer wall. Variant 1: external cladding made of fiber-reinforced concrete, δ_p_ = 20 mm; variant 2: cladding made of aluminum alloy, δ_p_ = 3 mm.

	Wall (concrete)	Insulation (mineral wool)	Mounting anchor	Stainless steel fastener with holes for heat flow extension, δ_f_ = 3 mm	Cladding (fiber-reinforced concrete) δ_p_ = 20 mm
ρ (kg m^−3^)	2400	40	7600	7600	2000
*λ* (W m^−1^ K^−1^)	2.5	0.036	15	4.26	1.5
c (J kg^−1^ K^−1^)	840	750	450	450	840

				Aluminum alloy fastener, δ_f_ = 4 mm	Cladding (fiber-reinforced concrete), δ_p_ = 20 mm
ρ (kg m^−3^)				2710	2000
*λ* (W m^−1^ K^−1^)	Values as in the line above			21.04	1.5
c (J kg^−1^ K^−1^)				1256	840

				Stainless steel fastener with holes for heat flow extension, δ_f_ = 3 mm	Cladding (aluminum alloy), δ_p_ = 3 mm
ρ (kg m^−3^)				7600	2710
*λ* (W m^−1^ K^−1^)	Values as in the line above			4.26	167
c (J kg^−1^ K^−1^)				450	1256.1

				Aluminum alloy fastener, δ_f_ = 4 mm	Cladding (aluminum alloy), δ_p_ = 3 mm
ρ (kg m^−3^)				2710	2710
*λ* (W m^−1^ K^−1^)	Values as in the line above			21.04	167
c (J kg^−1^ K^−1^)				1256	1256.1

Simulation-based calculations were performed in the COMSOL environment^18^ and included the determination of the temperature fields in the considered sections of multilayer walls, the heat transfer coefficient and the heat flux transferred through the multilayer wall. The simulations were based on the heat transfer equation described for the conditions determined by the relationship:

4 $$ \frac{\partial }{\partial x}\left( {\lambda_{x} \frac{\partial T}{{\partial x}}} \right) + \frac{\partial }{\partial y}\left( {\lambda_{y} \frac{\partial T}{{\partial y}}} \right) + \frac{\partial }{\partial z}\left( {\lambda_{z} \frac{\partial T}{{\partial z}}} \right) = - q $$

where T—temperature, λ_x_, λ_y_, λ_z_—thermal conductivity in x, y, z direction respectively, q—heat flux per unit volume.

A differential equation can have an arbitrary number of solutions as the integration constants can be arbitrarily selected. In order to find the correct solution, it is necessary to specify, among others, cross-border conditions, including:

- Initial conditions that define temperature distribution in the multilayer wall section at a selected moment of time (time zero).
- Boundary conditions that define heat transfer conditions at the outer surface of the multilayer wall section, i.e. ambient temperature (at some distance from the outside of the wall) 253 K and 293 K on the inside and respective and, accordingly, the convective heat transfer coefficients 8 W m^−2^ K^−1^; 25 W m^−2^ K^−1^. The numerical model consisted of 147,473 tetrahedral elements, which made the calculating effort quite significant but improved the reliability of the results. The error coarser mesh size was larger than for finer one. Numerical simulations were performed for a normal predefined mesh size. The methodology of sensitivity analysis was taken from^19^ and^20^.

**Figure 10:**
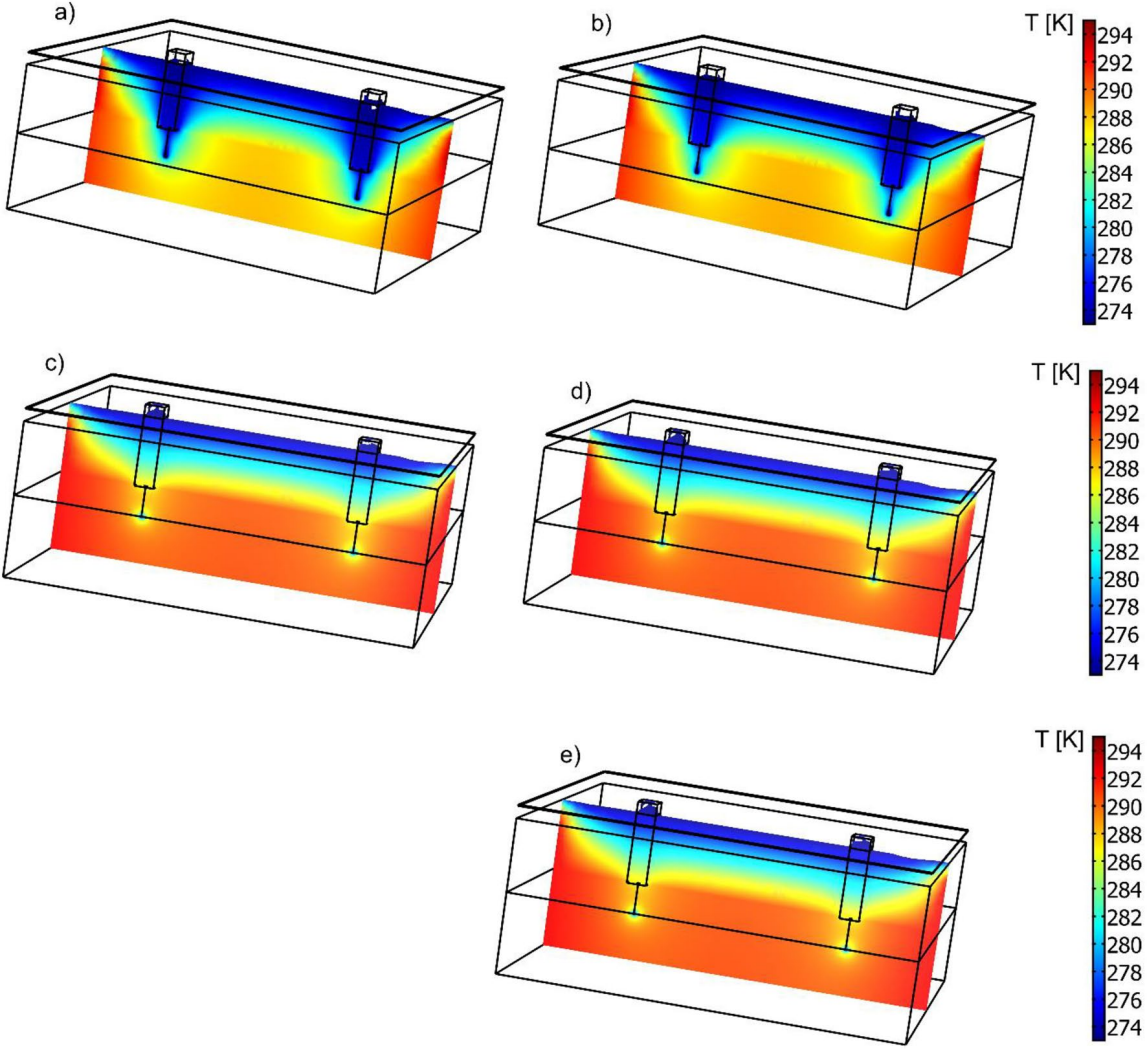
Computed temperature field in the multilayer wall cross-section cut by a plane passing through the fasteners. Cladding—fiber-reinforced concrete. Cases considered: (**a**) aluminum fastener, δ_f_ = 4 mm (**b**) aluminum fastener, δ_f_ = 2.75 mm, (**c**) stainless steel fastener δ_f_ = 3 mm, (**d**) stainless steel fastener with holes, δ_f_ = 3 mm, (**e**) stainless steel fastener δf = 2 mm.

Figure 10 shows an example of a cross-section of the multilayer wall fragment under analysis. The cross-section shows the temperature field with a color code to assess the low-temperature depth within the cross-section of a multilayer wall.

Analysis of the temperature field in the building wall shows a significant reduction in low-temperature regions when a corrosion-resistant steel fastener is used and a reduction in the low-temperature depth within the cross-section of the multilayer wall.

**Figure 11:**
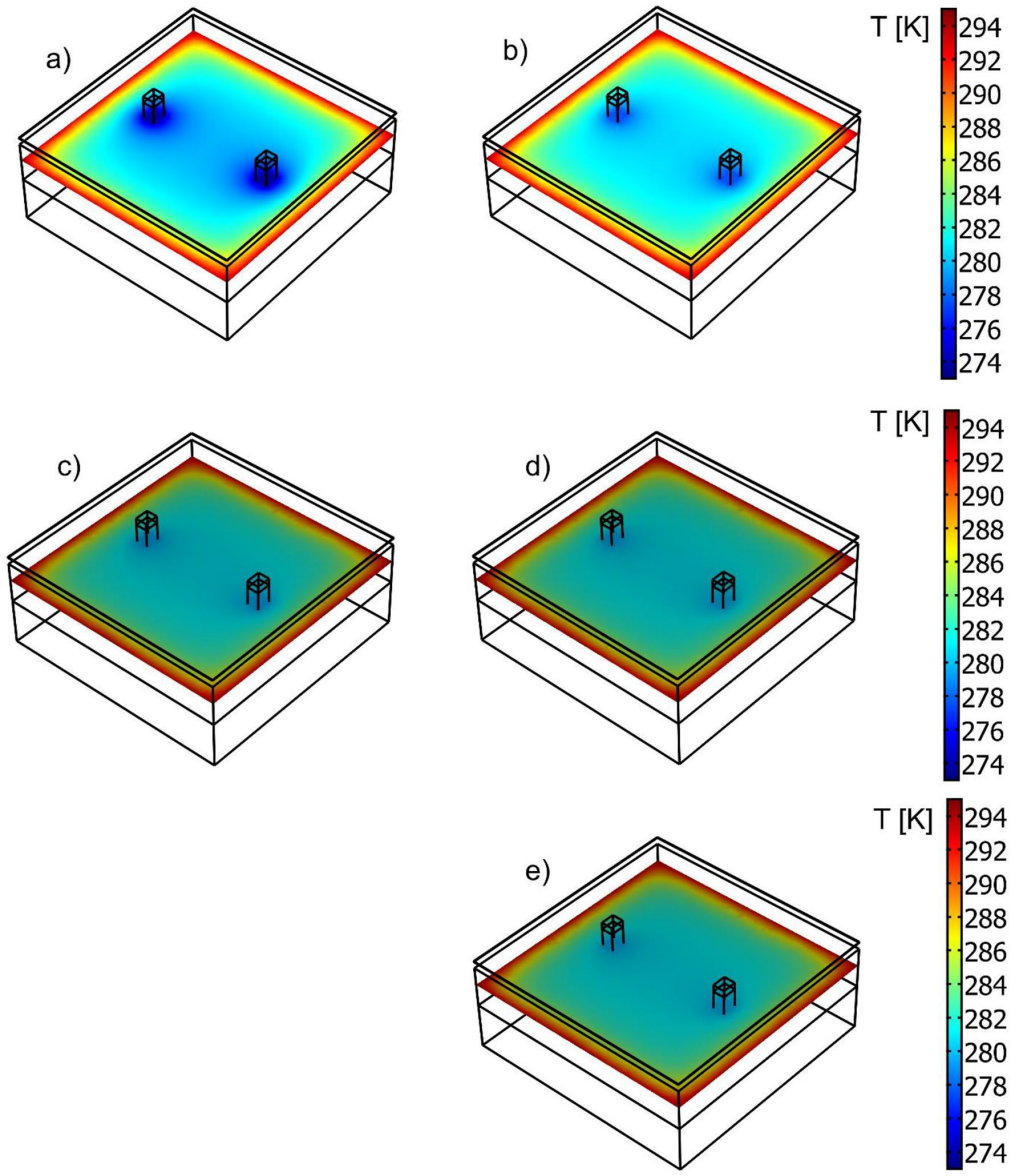
Computed temperature field in transverse cross-sections of the multilayer wall with an aluminum cladding. Cases considered: (**a**) aluminum fastener, δf = 4 mm (**b**) aluminum fastener, δ_f_ = 2.75 mm, (**c**) stainless steel fastener δ_f_ = 3 mm, (**d**) stainless steel fastener with holes, δ_f_ = 3 mm, (**e**) stainless steel fastener δ_f_ = 2 mm.

Figure 11 shows the temperature field in the transverse cross-sections of the wall fragments. The effect of the fastener location is visible, as are the markedly higher temperature gradients for the aluminum alloy fastener.

Table 5 compiles the final result of the simulation, i.e., the heat transfer coefficient and heat flux penetrating through the multi-layer wall. Analysis of the results in Table 5 shows a significant effect of the applied building fasteners on heat loss from the multi-layer wall. The thick mineral wool insulation is a major factor in heat conduction through building fasteners, significantly impacting heat loss.

**Table 5:** The results of the simulation calculations, including the heat flux penetrating 1 (m^2^) of the wall and the heat transfer coefficients for individual variants of the multilayer wall.

	Stainless steel fastener, δ_f_ = 2 mm	Stainless steel fastener, holes extending the heat flow path, δ_f_ = 3 mm	Stainless steel fastener,δ_f_ = 3 mm	Aluminum alloy fastener, δ_f_ = 2.75 mm	Aluminum alloy fastener, δ_f_ = 4 mm
Multilayer wall: concrete, mineral wool, fibre-reinforced concrete cladding, δp = 20 mm					
Heat flux (W m^−2^)	5.194	5.435	5.915	10.272	11.657
Overall heat transfer coefficient (W m^−2^ K^−1^)	0.130	0.135	0.148	0.257	0.291
Multilayer wall: concrete, mineral wool, aluminum cladding, δp = 3 mm					
Heat flux (W m^−2^)	8.011	8.446	9.324	20.546	24.112
Overall heat transfer coefficient (W m^−2^ K^−1^)	0.200	0.211	0.233	0.514	0.602

## Estimation of the heat transfer coefficient λ′ calculation uncertainty

Type A and type B measurement uncertainties for the effective thermal conductivity coefficient determination were calculated according to *the Guide to the expression of uncertainty in measurement*^21^. Type A standard uncertainty was calculated based on the statistical analysis of the measurements with standard deviation from the mean value as the basis. Type B standard uncertainty was determined based on the measurement uncertainties of the devices used in the experiment. The internal measuring system of the TDK Lambda power supply was used to measure the voltage, and the current supplied to the heater, for which the read maximum permissible measurement uncertainty was 0.05% of measured U and 0.3% of measured I. The heat transfer area was calculated on measured linear dimensions of the profile. The measurements were made with a caliper with a read uncertainty of 0.1 mm. To achieve a confidence level above 95%, we adopted the coverage factor kp = 2^21^ in statistical calculations. Table 3 presents the measurement uncertainty for the investigated building fasteners' effective thermal conductivity coefficient.

**Figure 12:**
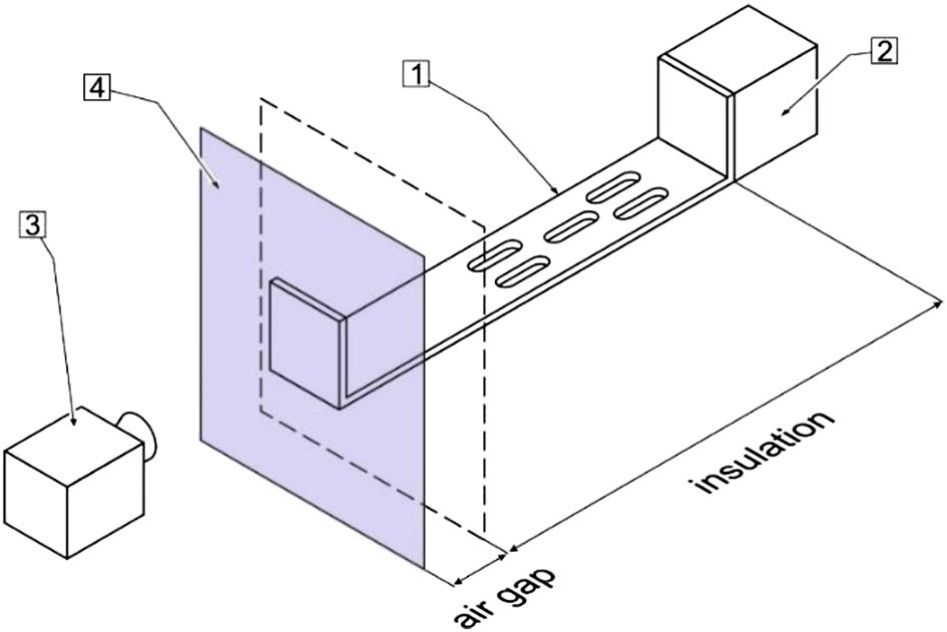
Schematic diagram of the experimental stand for digital model validation (not to scale). Description: 1—building fastener, 2—heater, 3—thermal imaging camera, 4—aluminum plate that simulates multilayer wall external cladding. Dimensions of the air gap and insulation are given in Fig. 9.

The proper performance of the numerical simulations should also include the validation stage^22^. To validate the numerical results, the actual operating conditions of the fasteners were simulated in the experimental stand, Fig. 12. It means the same temperature ranges and temperature differences between the internal and external sides of the wall assumed in the numerical simulation were recorded in the validating experiment.

Then, thermograms of the cladding surface connected to the fastener were made with the FLIR SC7600 thermal imaging camera. The temperature profile from the thermal imaging was consistent with the profile obtained from the simulation, which confirms that the measurement and the simulation techniques were done correctly.

**Figure 13:**
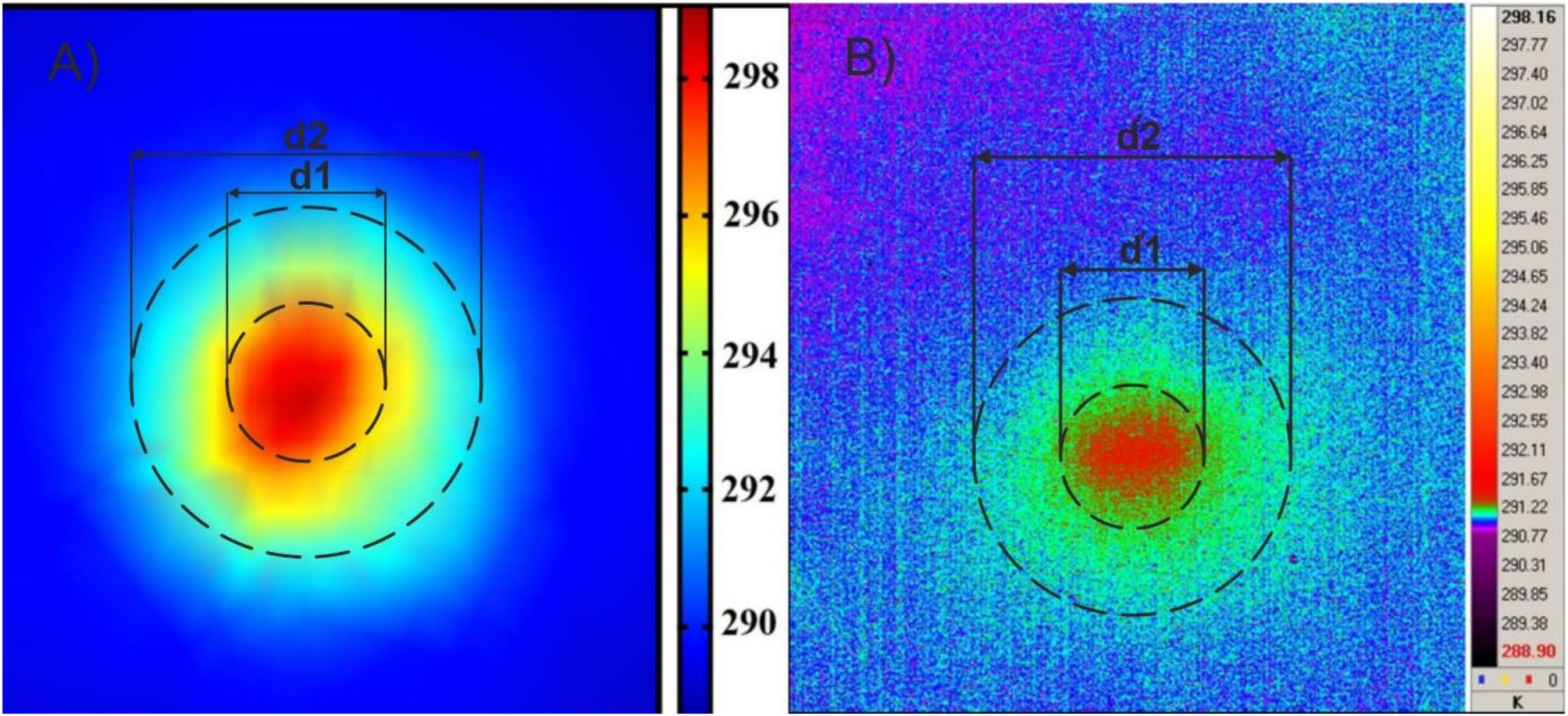
Temperature distribution around the interface between the fastener and the cladding at the conditions simulating natural temperatures of both elements: (**A**) numerical simulation, (**B**) recorded with thermal imaging camera. The temperature means were evaluated along the circles with diameters d_1_ and d_2_.

Figure 13 illustrates the temperature differences corresponding to the simulation model and the experimental thermograms. The temperature difference area studied was limited by two circles of diameters d_1_ and d_2_. The diameter d_1_, equal to 78 mm, corresponds approximately to that of the circle circumscribed around the fastener’s cross-section. Diameter d_2_ was twice the diameter d_1_ for simulation and validation.

The numerical results were validated by comparing the obtained values T_simul_ with temperature measurements Tmeasured in the corresponding conditions. A numerical model is considered well-validated if the relative difference between the results above is less than 10%^23^. The relative difference was calculated as follows:

$$ \varepsilon = \frac{{\left| {{\Delta }T_{measured} - {\Delta }T_{simul} } \right|}}{{{\Delta }T_{mesured} }} \times 100\% $$

Average values of the temperature simulated and measured at circles with diameters d_1_ and d_2_ are given in Table 6, where the value of relative difference ε is also shown.

**Table 6:** Comparison of numerical and experimental results.

	Temperature of area of the circle d1 (K)	Temperature of area of the ring (d2–d1) (K)
Measurement	291.38	291.16
Simulation	254.33	254.13
ε%	9.09%	

For all the investigated cases, the difference ε was less than 10%.

## Conclusions

The combination of numerical simulations and thermal imaging studies is an effective heat transfer investigation and building rationalization method. The experiment confirmed the accuracy of numerical simulation. Using stainless steel for fasteners is beneficial for heat transfer because steels have lower thermal conductivity than other traditional materials, e.g., aluminum alloys. It should be noted that the effective thermal conductivity coefficient defined in the article is not related to the cross-section of the fastener, but to the contact surface of the fastener with the heater and cooler surfaces. The tests showed a significant reduction of the effective thermal conductivity coefficient for a structure with holes extending the heat flow path. The results show that the fasteners whose walls have been milled have a lower heat transfer coefficient than those with solid walls. This effect can be attributed to the formation of labyrinth-like narrowings in the conductive material, which extend the heat flow path. Future attempts to reduce the effective thermal conductivity coefficient should focus on selecting appropriate steel alloys and optimizing the shape and number of holes extending the heat flux path. Attention should be paid to the fastener strength requirements. Proposed in the presented investigations structural method of improving the fastener thermal properties by extending the heat path flow is a new practice in building industry. The experimental data of the effective thermal conductivity for all tested fasteners proved the effectiveness of this new fastener design. These results recommend the new design of building fasteners with structural holes as the effective and economic way of improving their insulation parameters. The effective thermal conductivity used as an indicator of thermal performance of the fastener of is a new proposal in the subject literature. This indicator should be supported by verifying experimental tests in the way done in the paper.

## Data Availability

The datasets generated and/or analyzed during the current study are not publicly available due lack of consent from cooperating entities but are available from the corresponding author on reasonable request.

## List of symbols

A: Heat transfer surface (fastener/cooler interface surface) (m^2^)
c: Specific heat (J kg^−1^ K^−1^)
k_p_: Coverage factor
L: Fastener dimension in the heat flux flow direction (m)
I: Current (A)
Q: Heat flux (W)
T: Average temperature of the surface (K)
U: Voltage (V)
δ: Thickness (mm)
ε: Relative difference
λ′: Thermal conductivity coefficient (W m^−1^ K^−1^)
λ: Effective thermal conductivity coefficient (W m^−1^ K^−1^)
ρ: Density (kg m^−3^)

## Subscripts

cond: Heat conduction
el: Electrical
h: Heater
c: Cooler
f: Fastener
p: Panel
